# Is the information provided by large language models valid in educating patients about adolescent idiopathic scoliosis? An evaluation of content, clarity, and empathy: The perspective of the European Spine Study Group

**DOI:** 10.1007/s43390-024-00955-3

**Published:** 2024-11-04

**Authors:** Siegmund Lang, Jacopo Vitale, Fabio Galbusera, Tamás Fekete, Louis Boissiere, Yann Philippe Charles, Altug Yucekul, Caglar Yilgor, Susana Núñez-Pereira, Sleiman Haddad, Alejandro Gomez-Rice, Jwalant Mehta, Javier Pizones, Ferran Pellisé, Ibrahim Obeid, Ahmet Alanay, Frank Kleinstück, Markus Loibl, ESSG European Spine Study Group

**Affiliations:** 1 Department of Trauma Surgery, University Hospital Regensburg, Franz-Josef-Strauss-Allee 11, 93053 Regensburg, Germany; 2 Department of Spine Surgery, Schulthess Klinik, Zurich, Switzerland; 3 Spine Center, Schulthess Klinik, Zurich, Switzerland; 4 Spine Unit Orthopaedic Department, Hôpital Pellegrin Bordeaux, Bordeaux, France; 5 Dept. of Spine Surgery, Hôpitaux Universitaires de Strasbourg, Université de Strasbourg, Strasbourg, France; 6 Department of Orthopedics and Traumatology, Acibadem University School of Medicine, Istanbul, Turkey; 7 Spine Surgery Unit, Vall d’Hebron University Hospital, Barcelona, Spain; 8 Hospital Universitario Ramón y Cajal Spain, Madrid, Spain; 9 Spine Surgery, Royal Orthopaedic Hospital UK, Birmingham, UK; 10 Spine Surgery Unit, La Paz University Hospital, Madrid, Spain; 11 ESSG, European Spine Study Group, Barcelona, Spain

**Keywords:** Adolescent idiopathic scoliosis (AIS), Large language models (LLMs), Patient education, Spine surgery, Artificial intelligence (AI)

## Abstract

**Purpose:**

Large language models (LLM) have the potential to bridge knowledge gaps in patient education and enrich patient-surgeon interactions. This study evaluated three chatbots for delivering empathetic and precise adolescent idiopathic scoliosis (AIS) related information and management advice. Specifically, we assessed the accuracy, clarity, and relevance of the information provided, aiming to determine the effectiveness of LLMs in addressing common patient queries and enhancing their understanding of AIS.

**Methods:**

We sourced 20 webpages for the top frequently asked questions (FAQs) about AIS and formulated 10 critical questions based on them. Three advanced LLMs—ChatGPT 3.5, ChatGPT 4.0, and Google Bard—were selected to answer these questions, with responses limited to 200 words. The LLMs’ responses were evaluated by a blinded group of experienced deformity surgeons (members of the European Spine Study Group) from seven European spine centers. A pre-established 4-level rating system from excellent to unsatisfactory was used with a further rating for clarity, comprehensiveness, and empathy on the 5-point Likert scale. If not rated 'excellent', the raters were asked to report the reasons for their decision for each question. Lastly, raters were asked for their opinion towards AI in healthcare in general in six questions.

**Results:**

The responses among all LLMs were ‘excellent’ in 26% of responses, with ChatGPT-4.0 leading (39%), followed by Bard (17%). ChatGPT-4.0 was rated superior to Bard and ChatGPT 3.5 (*p* = 0.003). Discrepancies among raters were significant (*p* < 0.0001), questioning inter-rater reliability. No substantial differences were noted in answer distribution by question (*p* = 0.43). The answers on diagnosis (Q2) and causes (Q4) of AIS were top-rated. The most dissatisfaction was seen in the answers regarding definitions (Q1) and long-term results (Q7). Exhaustiveness, clarity, empathy, and length of the answers were positively rated (> 3.0 on 5.0) and did not demonstrate any differences among LLMs. However, GPT-3.5 struggled with language suitability and empathy, while Bard’s responses were overly detailed and less empathetic. Overall, raters found that 9% of answers were off-topic and 22% contained clear mistakes.

**Conclusion:**

Our study offers crucial insights into the strengths and weaknesses of current LLMs in AIS patient and parent education, highlighting the promise of advancements like ChatGPT-4.o and Gemini alongside the need for continuous improvement in empathy, contextual understanding, and language appropriateness**.**

**Supplementary Information:**

The online version contains supplementary material available at 10.1007/s43390-024-00955-3.

## Introduction

Generative artificial intelligence (AI), notably through AI-driven platforms like chatbots, has revolutionized the landscape of patient education by delivering personalized, easily comprehensible content that simplifies complex medical topics [1, 2]. These tools are integral in enhancing the patient-physician relationship by providing real-time, tailored educational resources, and facilitating more informed patient-level decision-making [3, 4]. Patients are increasingly likely to consult large language models (LLMs) during their internet searches on medical conditions, making it essential to ensure the accuracy of the information provided by these models [5, 6]. The recent European Union (EU) AI Act, enforcing strict AI regulation, emphasizes the importance of careful oversight in healthcare applications [7]. The introduction of widely accessible chatbots, such as ChatGPT, which employs advanced GPT3.5 and GPT4 LLMs, led to many different applications including the use of these models for private patient education [8, 9].

Adolescent idiopathic scoliosis (AIS) has a significant impact on patients’ lives, presenting physical challenges like pain and reduced mobility, and psychological issues, such as body image concerns, thus making patients with AIS likely to search for information about their life-changing condition from multiple sources, including LLMs. Communication with AIS patients and families should address the emotional impact and the condition’s long-term management [10]. Treatment aims to balance physical correction with patient expectations about appearance and quality of life. However, the current literature does not conclusively favor any specific treatment method over others for severe AIS [11]. Surgical interventions pose risks and necessitate careful consideration of future growth in younger patients [12, 13]. Psychologically, AIS can lead to significant stress, anxiety, and body image issues [14, 15]. Adolescents with AIS may feel self-conscious about their appearance or the need to wear a brace, leading to social isolation or depression [16]. Aesthetics are a vital consideration in treatment, as spine curvature visibility can affect self-perception [17]. Given these complexities, healthcare providers are dedicated to offering clear, empathetic communication, and comprehensive education about AIS, and recent literature suggests that AI could serve as an assistive tool in enhancing empathy, compassion, shared decision-making, and healthcare trust [18].

Concrete evidence of AI’s impact on patient-surgeon relationships is limited, especially regarding its implementation in patient-centered care. We hypothesized that different, publicly available LLMs can provide comparable and valid answers to patient questions on AIS. The objective of this study was to evaluate the validity, clarity, and empathy of information provided by LLMs in hypothetically educating patients and parents about AIS , through responses to 10 frequently asked questions (FAQs). We employed a structured assessment by first identifying FAQs through an internet search, then having these questions answered by LLMs, and finally having the LLMs’ responses evaluated by a panel of members of the European Spine Study Group (ESSG) to determine their quality and accuracy.

## Methods

### Identification of relevant FAQs

To identify FAQs of general patient interest, a comprehensive Google search was conducted using the search term: ´ *frequently asked questions OR FAQ AND adolescent idiopathic scoliosis OR AIS OR scoliosis AND growth OR Adolescent*´ yielding approximately 162,000 results within 0.46 s (October 20th, 2023; region:Germany). For this study, the first 20 Google hits were checked and the following inclusion and exclusion criteria were applied (Table 1).

**Table 1:** Inclusion and exclusion criteria for questions

Inclusion criteria	Exclusion criteria
Published after January 1st, 2017	Non-generalizable information e.g., provider or implant-specific details
Published in English language	Emphasis on non-spine-surgical aspects, e.g., anesthesiologic information
Information presented in FAQ or Q&A sections	

The search results were screened by the authors using these criteria. From the array of sources available, a pool of FAQs from thirteen institutions (Suppl. Material 1) was used to identify the most recurrent FAQs. In addition, ChatGPT-4 was directly engaged with the prompt (October 20th, 2023; region: Germany): “Suggest a list of the 20 most common frequently asked patient questions about adolescent idiopathic scoliosis (AIS)” to generate a list of questions relevant to our study.

This two-step approach resulted in a consolidated pool of 135 questions about AIS. From this pool, the 10 most frequently recurring topics were identified and ranked (Table 2). The authors carefully reviewed this ranked list and crafted 10 new questions, synthesizing the essence of these topics, which were then presented to the three LLMs for evaluation (Table 3). In instances of discord, the authors collaboratively agreed on a consensus in the formulation of the final question set.

**Table 2:** The Ranking of the most frequent 10 topics about AIS derived from online sources with FAQs

Ranking	Topic	Sample questions from the online sources	Frequency
1	Definition of AIS	What is Adolescent Idiopathic Scoliosis (AIS)? Are there different types of scoliosis? What is the difference between idiopathic scoliosis and other types of scoliosis?	15
2	Diagnosis of AIS	How is AIS diagnosed? How Useful Is Physical Examination in Detecting Clinically Significant Scoliosis? How will the doctors check if I have scoliosis?	14
3	Treatment options	What are the treatment options for AIS? What Treatments Are Effective? Is Scoliosis Treatable?	14
4	Causes and mechanisms of AIS	What causes AIS? Does my child´s bad posture cause scoliosis? Do sports activities or heavy backpacks cause scoliosis? Is scoliosis related to an injury?	13
5	Pathophysiology and progress	How does AIS progress? How do we estimate remaining growth, and thus the likelihood of scoliosis progression? Does Idiopathic Scoliosis Get Worse?	9
6	Restrictions after surgery	How will the rods affect my spine’s mobility and my activities? Can I safely deliver a baby in the future after scoliosis surgery? When can I return to my sports, dance and other physical activities?	9
7	Prognosis and outcome	What is the long-term outlook for individuals with AIS? What is the outcome of treatment of scoliosis? What health problems might I have later in life as a result of scoliosis? Will my child be able to live a normal life?	6
8	Postsurgical aftercare/follow-up	Will I have pain after surgery? Will I need a brace after surgery? How often should follow-up appointments be scheduled?	6
9	Aesthetics	Will I have a hump on my back when I get older? Will my waist, back and shoulders still be uneven, even after surgery? How can I make my scar as minimal as possible?	6
10	Symptoms and clinical presentation	What are the signs and symptoms of AIS? Does scoliosis cause back pain? How do I know if my child has scoliosis?	5

**Table 3:** 10 FAQs about AIS

1	What distinguishes Adolescent Idiopathic Scoliosis from other scoliosis types, and are there different forms?
2	How is AIS diagnosed, and what role do screening, imaging, and physical examination play in detecting it?
3	Can you summarize the treatment options for AIS and their indications and overall effectiveness?
4	What are the primary causes of AIS, and could posture, sports, or carrying heavy items have contributed to it?
5	How is the progression of AIS estimated, especially in relation to my child's growth and how likely is it?
6	What restrictions on physical activity and future life events like pregnancy can we expect after scoliosis surgery?
7	What long-term outcomes should we anticipate for my child with AIS, including potential health issues and lifestyle impacts?
8	What does aftercare involve, and how often are follow-up visits needed post-surgery for AIS?
9	Will surgery correct the cosmetic concerns of AIS, like uneven shoulders or back humps, and what can be done to minimize the visibility of their scar?
10	What are the key symptoms and the clinical presentation of AIS, and is back pain a significant indicator?

Next, the questions were submitted to the publicly accessible AI chatbot ChatGPT3.5 through its online portal (https://chat.openai.com/chat) on October 21st, 2023, (Answer Set #1). Second, the questions were relayed to ChatGPT 4.0 (Answer Set #2). Third, the identical questions were presented to Google’s chatbot "Bard" (https://bard.google.com/chat) on the same date (Answer Set #3). All three LLMs were prompted with the same subsequent text used before each question:

“*Act as an expert spine surgeon who is up to date with the latest scientific research and has years of experience counseling patients with empathy and clarity. Provide comprehensive and easily understandable answers to the following question about adolescent idiopathic scoliosis! Ensure the responses are timely, incorporate the most recent advancements, and address potential concerns patients and parents might have. Limit your answer to 200 words and focus on the most important aspects to ensure patient and parent information:* (…)”

For each question, a new window of the respective chatbot was created to avoid any biases from the prior questions ("context bias/conversation drift"). After the answers were generated, they were recorded verbatim in our database.

### Raters and rating of LLM responses

The LLMs responses (Suppl. Material 2), recorded after the first query without repetition, underwent strict evidence-based evaluation using a pre-reported rating system [19]. Responses were rated as either ‘excellent’ (no clarification needed), ‘satisfactory with minimal clarification’ (factually correct but lacking detail or nuance), 'satisfactory with moderate clarification' (containing outdated or irrelevant information), or 'unsatisfactory' (prone to misinterpretation due to outdated or overly generic data). Satisfactory responses were factually sound butwould  require some explanation according to the raters. The evaluative framework was augmented with the subsequent four inquiries (Table 4), wherein participants were provided with a 5-point Likert scale extending from 'I strongly disagree' to 'I strongly agree' [20]. The participants were asked to answer these four inquiries referring to each of the three answer sets.

**Table 4:** Supplementary evaluation criteria for each data set

No	Evaluation criteria
1	The overall content of all answers is comprehensive and covers all necessary aspects
2	The answers are easy to understand and are communicated clearly
3	The answers address patient concerns empathetically and professionally
4	The overall length and detail of each answer are appropriate for the target audience

The answer set for each LLM was provided to the raters using the online Google Forms application. Raters were blinded to the different LLMs. Each response was subjected to a rigorous evaluation by ten independent raters from the European Spine Study Group consisting of a group of experienced spinal deformity surgeons from 7 centers that brings together the knowledge and experience of renowned clinicians and researchers, active in the field of spinal deformity. Finally, the raters were presented with seven inquiries aimed at eliciting their preference for the best set of three responses, followed by additional questions designed to collect their general perspective on the utilization of AI tools in patient care (Table 5). A 5-point Likert scale has been used to answers these questions.

**Table 5:** Questions for final evaluation and general opinion

1. In your opinion, which of the above 3 sets contained the highest quality answers and answered the 10 FAQs most appropriately and professionally?
2. In general, have the above responses met your expectations of the performance of currently available LLMs?
3. Based on your experience with the scored responses above, would you consider integrating LLM or AI-based patient information into any aspect of your clinical practice in the future?
4. In your opinion, how could the utilization of LLMs improve the patient experience, especially in streamlining the information process before and after surgical procedures?
5. Do you think the integration of LLMs in healthcare could alleviate some of the workload on medical staff, particularly in providing initial information to patients?
6. How do you foresee the role of AI/LLMs in optimizing the patient-physician relationship and communication, particularly in ensuring patients are well-informed and prepared for their surgical procedures?
7. What is your general attitude toward the development of AI/LLMs in healthcare?

### Statistical analysis

Data are presented using absolute values, percentages, mean and standard deviations (SD) for descriptive purposes. The interrater reliability was assessed using Fleiss Kappa. Chi-square tests (*χ*2) were applied to test differences in ratings among LLMs, raters and questions and the differences in the reasons for not satisfying the responses. A Friedman test was applied to test differences among LLMS in exhaustiveness, clarity, empathy, and length. All statistical procedures were performed using GraphPad Prism 9.5.1. The level of statistical significance was set at *p* < 0.05.

## Results

**Fig. 1:**
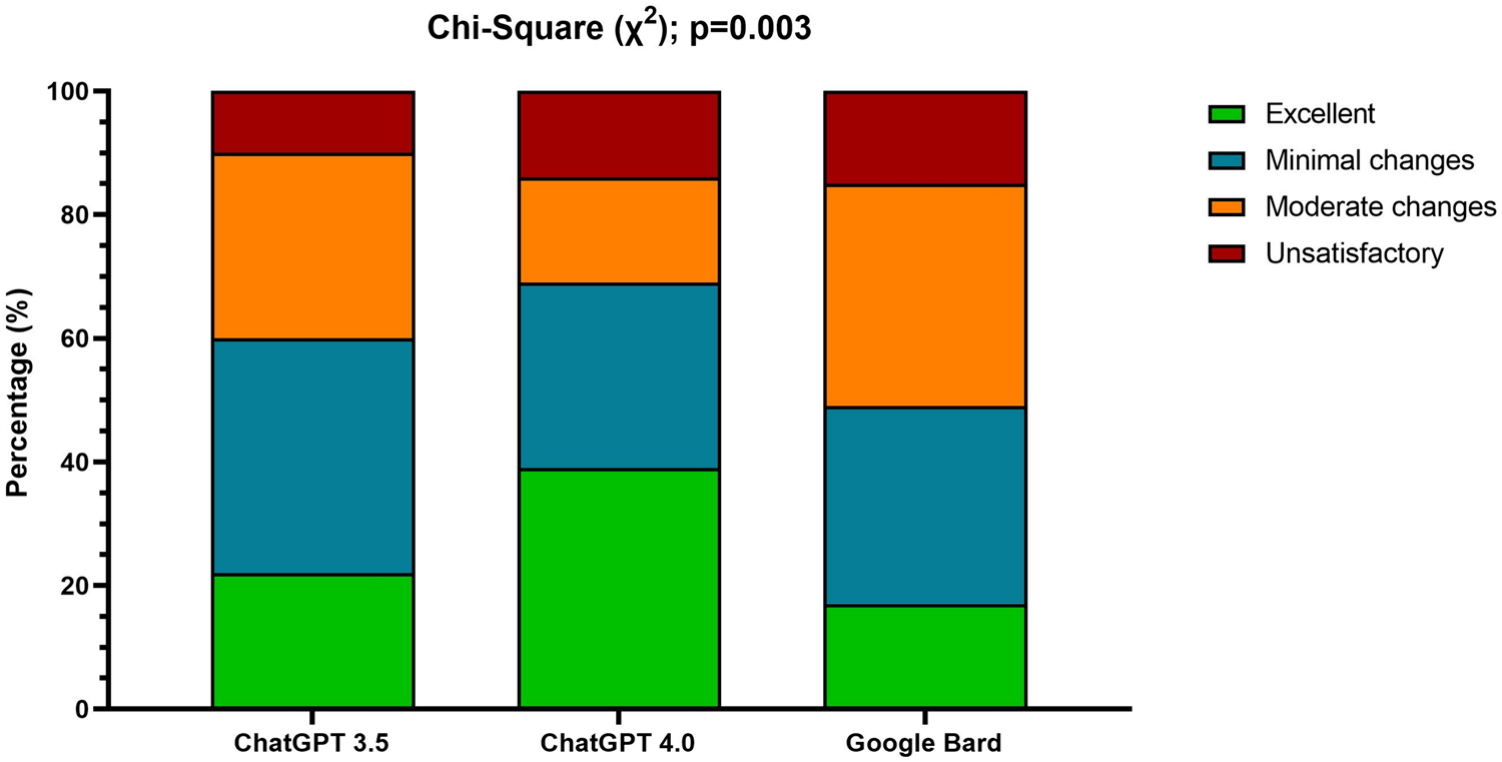
Histograms with the rating distribution, expressed in percentages, for ChatGPT 3.5, ChatGPT 4.0, and Google Bard. The *χ*2 highlighted a significant difference among LLMs (*p* = 0.003)

The pooled performance of three LLMs showed 26% of responses were rated ‘excellent’, 33% 'satisfactory with minimal clarification', 28% ‘satisfactory with moderate clarification’, and 13% were ‘unsatisfactory’. For ChatGPT-3.5, 22% of responses were rated ‘excellent’, 38% were ‘satisfactory with minimal clarification’, 30% were ‘satisfactory with moderate clarification’, and 10% were ‘unsatisfactory’. ChatGPT-4.0 saw a high proportion of ‘excellent’ responses at 39%, with only 14% ‘unsatisfactory’, but 17% still required moderate clarification and 30% minimal. Bard had the highest percentage of ‘unsatisfactory’ responses at 15%, most answers needing ‘moderate clarification’ (36%), 32% ‘satisfactory with minimal clarification’, and the least percentage of ‘excellent’ responses (17%). Surgeons rated the answers of ChatGPT-4.0 superior, notably outperforming Bard with statistical significance (p = 0.003, Fig. 1).

**Fig. 2:**
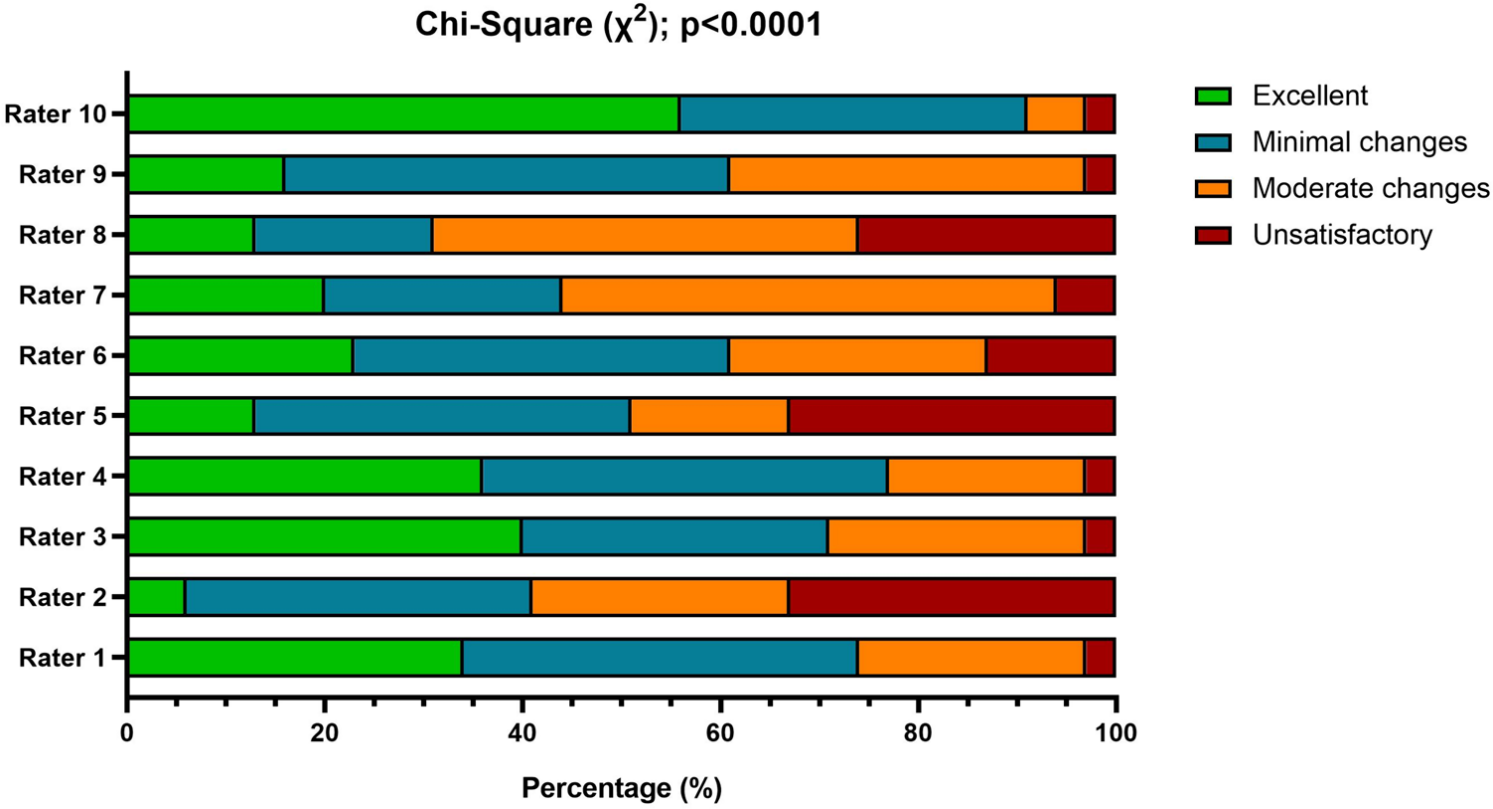
Histograms with the rating distribution, expressed in percentages, for each rater. The *χ*2 highlighted a significant difference (*p* < 0.0001) in the rating distribution among raters

Significant discrepancies were observed among raters evaluating each LLM separately (ChatGPT3.5: *p* < 0.05; ChatGPT4.0: *p* < 0.0001; Google Bard: *p* < 0.0001) and all LLMs pooled (*κ* = 0.23; *p* < 0.0001; Fig. 2).

**Fig. 3:**
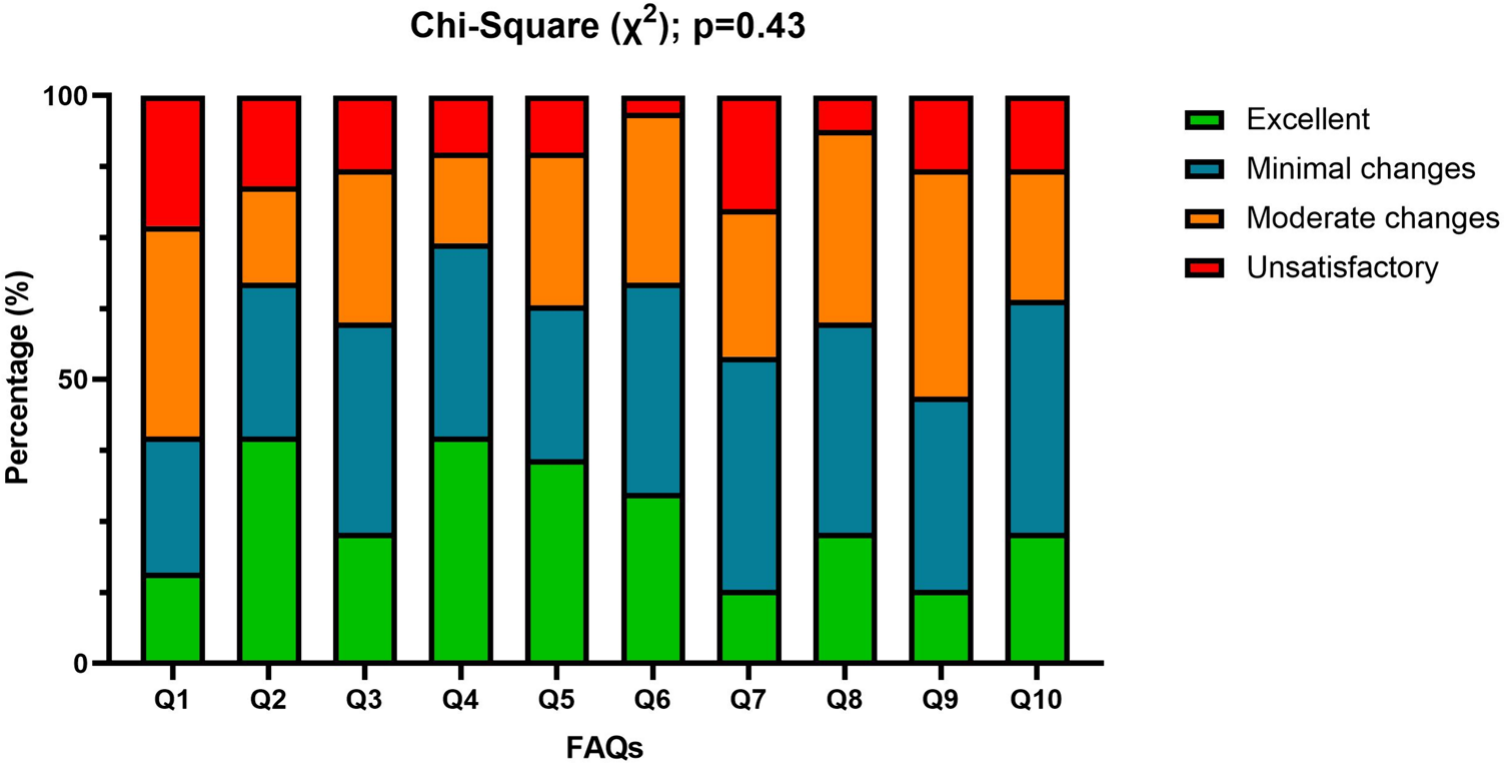
Histograms with the ratings distribution, expressed in percentages, for each FAQ, from Q1 to Q10. The *χ*2 did not show a significant difference (*p* = 0.43) in the rating distribution among questions

**Fig. 4:**
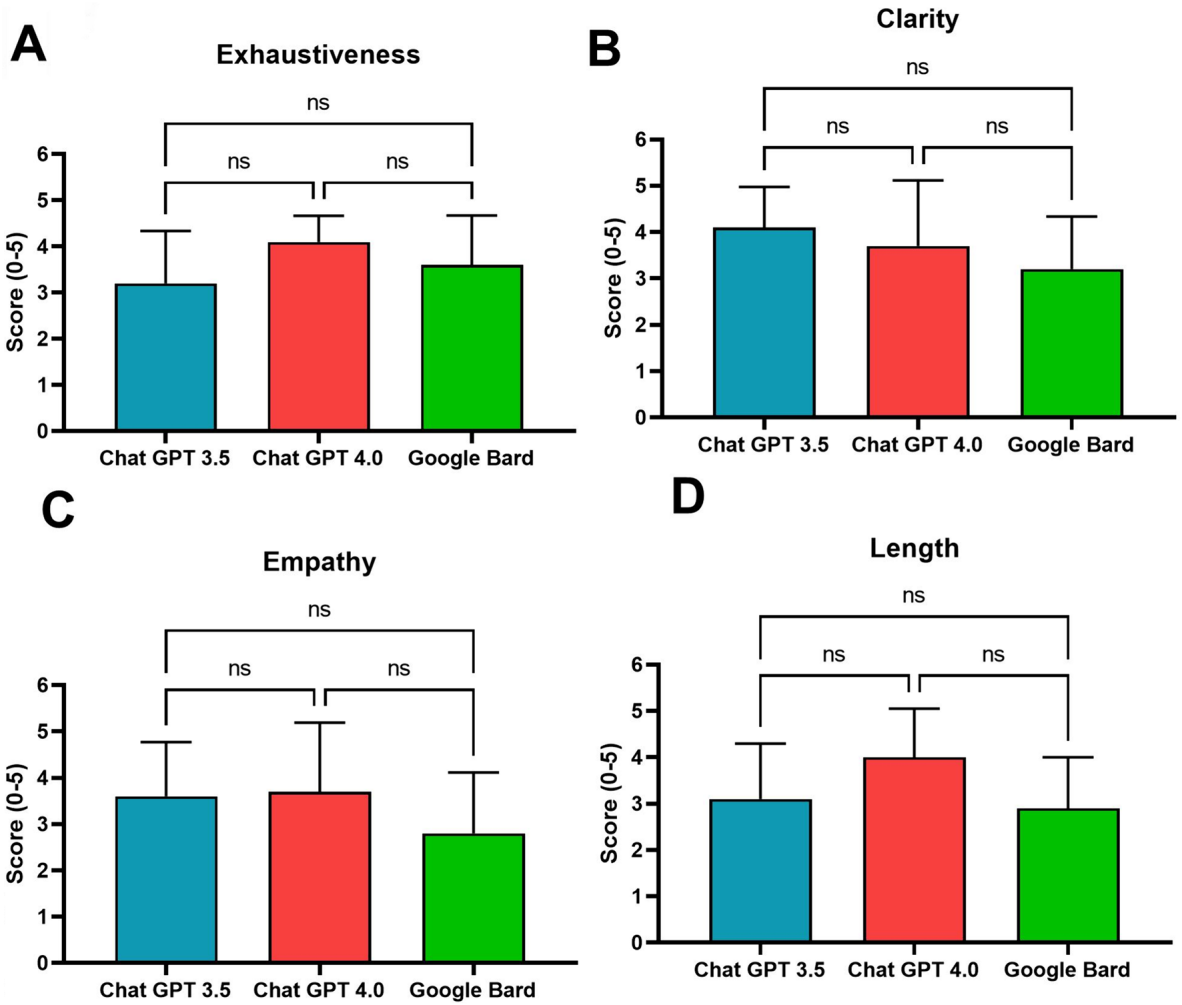
Histograms with mean and SD for the scores reported by raters, on a Likert scale from 1 to 5, of exhaustiveness (panel A), clarity (panel B), empathy (panel C), and length of the answers (panel D). The Friedman test did not show any significant differences among LLMs. *Legend*: ns, non-significant

No significant differences were observed in the distribution of ratings among questions (*p* = 0.43, Fig. 3). In total Q2 (Diagnosis) and Q4 (Causes/Pathophysiology) received high percentages of 'excellent' ratings (40% each). The lowest rates of ‘excellent’ ratings were seen in Q7 (Long-term outcome) and Q9 (Surgical correction of cosmetic concerns) (13% each). The highest rates of ‘unsatisfactory’ ratings were found in Q1 (Definition) and Q7 (23% and 20%; Fig. 4). ChatGPT-3.5’s best-rated response was for Q5 with 50% ‘excellent’ ratings, while the worst were for Q9 and Q10, both at only 10% ‘excellent’. ChatGPT-4.0 excelled in Q2, Q4, and Q6 with 70%, 60%, and 60% ‘excellent’ ratings respectively, and had lower ratings with Q7, Q8, and Q9. Bard performed best on Q4, Q5, and Q10, with each 30% ‘excellent’ ratings, but had its lowest ratings for Q1, Q2, and Q3, with 40%, 20%, and 20% ‘unsatisfactory’ ratings and 0%, 10% and 10% ‘excellent’ ratings, respectively.

Exhaustiveness, clarity, empathy, and length of the answers were rated > 3.0 for each LLM. The Friedman test did not show any significant differences among LLMs (Fig. 4).

**Fig. 5:**
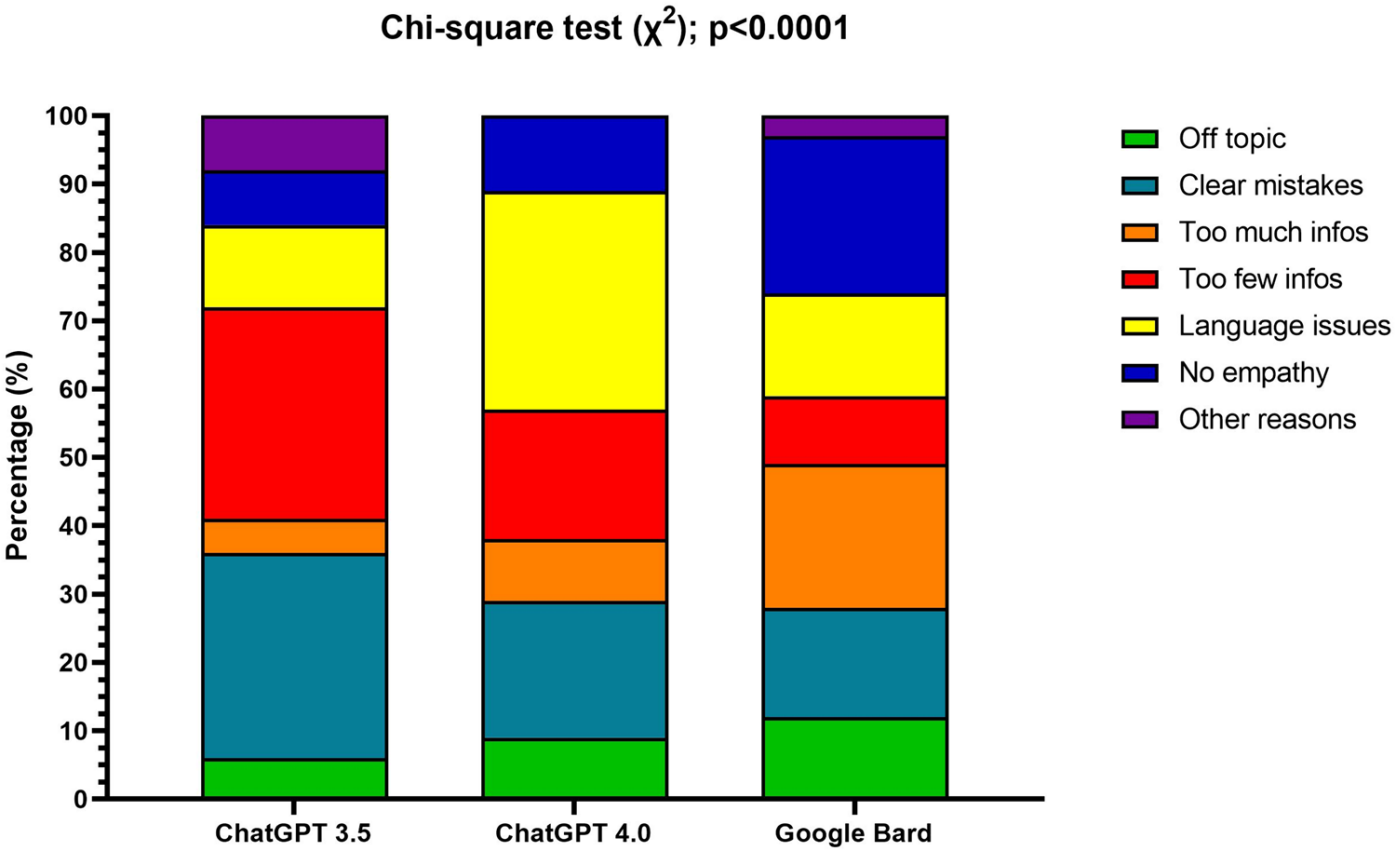
Histograms showing the distribution of the reasons, reported by raters, for not satisfying the responses for ChatGPT 3.5, ChatGPT 4.0, and Google Bard separately. The *χ*2 showed a significant difference (*p* < 0.0001) among LLMs

From the answers rated worse than ‘excellent’ (ChatGPT-3.5: 78.0%; ChatGPT-4.0: 61.0%; Bard: 83%) the raters found ChatGPT-3.5´s answers to contain ‘clear mistakes’ in 30%and 32% responses were found to contain ‘too little information’, while ChatGPT-4.0 presented a lower rate of answer comprising 'too little information' and 'clear mistakes' with only 20% in both categories. Bard had the lowest rate of responses with ‘clear mistakes’ (16%), but the highest rate of responses deemed ‘too informative’ (21%I. Overall, Bard's answers were considered less empathetic (24%), compared to ChatGPT-3.5 and ChatGPT-4.0 (both 8–11%). Appropriate wording was noted in 33% of ChatGPT-4.0´s answers, and even lower in ChatGPT-3.5's (12%) and Bard´s (15%), respectively (Fig. 5; *p* < 0.0001). Overall, the raters found 9% of the pooled answers (that were rated less than 'excellent') off-topic, 22% of answers cited clear mistakes, 12% of answers contained too much information, 21% of answers comprised too few details, and 18% contained language issues unsuitable for patients, with an additional 14% of the answers lacking empathy.

Seven raters endorsed ChatGPT 4.0 for providing the highest quality and most professional responses to the 10 FAQs, while three raters favored ChatGPT 3.5; and Google Bard received no votes. The ratings in Table 6 correspond to the evaluative questions of Table 5 and display a range of mean scores from 3.4 to 4.1 for Q2 to Q7. Raters exhibited a generally positive outlook on the role of LLMs in enhancing patient experience through streamlined pre- and post-surgical information processes. Their stance on the incorporation of LLMs in healthcare was favorable. However, they expressed skepticism regarding the capacity of LLMs to significantly reduce medical staff workload, especially in the initial patient information provision.

**Table 6:** Ratings for the questions on the final evaluation and general opinion by raters

Ratings for the questions on the final evaluation and general opinion by raters	
Q1	n = 3: ChatGPT 3.5 n = 0: Google Bard n = 7: ChatGPT 3.5
Q2	4.2 ± 0.4
Q3	4.2 ± 0.7
Q4	4.0 ± 0.6
Q5	4.2 ± 0.7
Q6	4.3 ± 0.5
Q7	4.8 ± 0.4

Data are reported as mean ± SDs

## Discussion

Our study highlights publicly accessible LLMs’ ability to deliver nuanced, accurate responses to AIS queries, demonstrating AI’s promise and current limitations for patient education. Performance varied among LLMs like Bard and ChatGPT versions, with many answers lacking clarity and some unsatisfactory. This inconsistency across AIS-related questions points to the need for enhanced accuracy and interaction. The goal is to combine detailed knowledge with human-like empathy, improving AI’s grasp of human thought and emotion in healthcare communication, especially for the complex, emotionally charged context of AIS patient and family interactions. Key aspects include the need for educating parents to enhance AIS recognition, combined with the necessity of professional screening; providing diverse and specific information tailored to individual needs, and setting realistic expectations for post-treatment activities [21].

The important implication of providing easily accessible and accurate information about the diagnosis, causes and pathophysiology of AIS is underscored by findings of a cross-sectional study by de Groot et al. They examined the effect of educating parents to recognize scoliosis, especially in countries where the responsibility for detection has shifted from healthcare professionals to parents, leading to more late presentations: 100 parents assessed two series of cases for scoliosis, both before and after receiving educational information, resulting in a slight but significant increase in sensitivity for detecting scoliosis [22]. The study demonstrates that educating parents enhances their ability to identify scoliosis without increasing false positives, yet it cannot match the sensitivity of professional screening, underscoring the irreplaceable role of professional diagnosis. Parents and patients prefer attending surgeons to personally explain the consent, often requiring multiple explanations with visual aids: Chan et al. aimed to understand parents’ and patients’ perceptions of the informed consent process before posterior spinal fusion for adolescent idiopathic scoliosis [23]. Despite understanding and signing the informed consent, patients and parents still held surgeons accountable for complications, especially concerning risks like death, neurological deficit, and screw-related injuries [23]. Innovative tools like Chat-Orthopedist, based on retrieval-augmented LLMs, have been developed to aid AIS patients and families in preparing for meaningful discussions with clinicians [24]. The authors introduced a shared decision-making tool for AIS patients and families, utilizing a retrieval-augmented ChatGPT that integrates an external AIS knowledge base for accurate responses aiming to enhance clinical visits and treatment decisions through interactive learning and continuous human evaluations for system refinement [24]. LLMs could play a pivotal role in supplementing the educational needs of parents and patients, providing accessible and accurate information about AIS diagnosis, causes, and pathophysiology.

The lowest rates of ‘excellent’ ratings in Q7 (Long-term outcome) and Q9 (Surgical correction of cosmetic concerns) suggest that LLMs face difficulties with questions requiring nuanced understanding, long-term prognostic predictions, and aesthetic judgments. These areas might demand a deeper level of expertise and understanding of patient-specific contexts, which are challenging for current LLMs. The challenges faced by LLMs as highlighted in our study, align with the current literature emphasizing the intricate information needs of AIS patients and their families. A study by Wellburn et al. assessed the information needs of AIS patients and their families and stressed the necessity for accurate, individualized, and easily understandable information materials [25]. Their primary need for information centered on the cause and prognosis of the condition, and there were varying opinions on the quality of the information they received [25]. These findings highlight the need for a holistic approach in AIS care, one that goes beyond clinical treatment to encompass empathetic communication and support for both patients and their families.

AI and machine learning hold promise for transforming spine care with data-driven insights for better patient selection and education, surgical planning, and personalized recovery strategies [26, 27]. Notable, medical misinformation and patient ‘over-information’ are still major risks and issues [28, 29]. In the current study, raters found clear mistakes in 22% of all answers, among the answers rated worse than 'excellent'. ChatGPT-4.0 led with 39% ‘excellent’ ratings, surpassing ChatGPT-3.5’s 22% and Bard's 17%, indicating its superior performance in query responses. In a study by Ali et al. assessing the performance of ChatGPT-3.5, ChatGPT-4, and Google Bard on a neurosurgery oral boards preparation question bank, ChatGPT-4 significantly outperformed the others with a score of 82.6% [30]. The study highlighted ChatGPT-4’s superior accuracy in higher-order management case scenarios and lower rates of incorrect or irrelevant responses compared to ChatGPT-3.5 and Bard. The variations in performance between different LLMs underscore the importance of choosing the right AI tool for specific educational purposes. However, the superior performance of ChatGPT-4.0, as rated by surgeons and demonstrated in comparative studies, indicates a positive trend in the evolution of AI capabilities, but also highlights the necessity for continuous updates and improvements in these models to ensure they remain relevant and accurate.

The lack of significant differences in the distribution of ratings across questions in our study suggests a relatively consistent performance of the LLMs across various question types. However, the variation in the percentage of ‘excellent’ and ‘unsatisfactory’ ratings for specific questions indicates that certain topics were better addressed by the LLMs than others. Both Q2 (Diagnosis) and Q4 (Causes/Pathophysiology) received high percentages of ‘excellent’ ratings (40% each). This indicates that the LLMs are particularly adept at handling questions involving factual recall and basic medical understanding, areas where structured and well-defined information is available. The ability of LLMs to handle complex medical queries effectively is largely dependent on their exposure to and training in relevant medical data. Domain specificity is key to accuracy in specialized fields [31]. LLMs trained or fine-tuned on specific domains can process and recall factual information more accurately, as seen in the case of ClinicalGPT, which is a language model specifically designed and optimized for clinical scenarios in healthcare. [32]. The most recent introduction of Google’s Med-PaLM 2 into the medical field suggests a potential advancement in the capabilities of LLMs for patient education [33].

## Limitations

Our study must consider the rapid progression of LLMs, which could make our findings less relevant due to their enhanced capabilities in specific domains like medicine. The opacity in how LLMs refine responses, especially on sensitive issues, complicates understanding their source—AI or human adjustment. The shift to paid access for ChatGPT 4, contrasting with the open access of its predecessors, affects comparability and the tradition of open-source use. Our one-sided empathy assessment and lack of interactive feedback limits evaluating the LLM’s comprehensive understanding and emotional engagement. Furthermore, the study’s reliance on a few raters and no standardized evaluation approach warrants a careful interpretation of the results. A potential pro-LLM bias among raters suggests future studies should include a comparison to human responses for a more balanced analysis. Additionally, future studies should include direct patient feedback to assess whether the LLMs’ responses adequately address patients' questions and concerns. A mixed-methods approach, incorporating both quantitative and qualitative evaluations from patients and physicians, would provide a more holistic understanding of the LLMs’ performance.

## Conclusion

We provide valuable insights into the capabilities and limitations of current, publicly available, and commonly used LLMs in the context of patient and parent education for AIS. While advancements like ChatGPT-4.0 show promise, there is a clear need for ongoing improvement, particularly in areas such as empathy, contextual understanding, and appropriate wording.

## Supplementary Information

Below is the link to the electronic supplementary material.

Supplementary file1 (DOCX 34 KB)

Supplementary file2 (DOCX 38 KB)

## Data Availability

The data supporting the findings of this study are available from the corresponding authors upon reasonable request.
