# Supplementary material for: Is the information provided by large language models valid in educating patients about adolescent idiopathic scoliosis? An evaluation of content, clarity, and empathy: The perspective of the European Spine Study Group
Source: Spine Deform. 2024 Nov 4;13(2):361–72. doi: 10.1007/s43390-024-00955-3 (PMC11893626; doi:10.1007/s43390-024-00955-3)
Supplement: Supplementary file 1 — Supplementary file1 (DOCX 34 KB) [file 43390_2024_955_MOESM1_ESM.docx]

| **Supplementary Material 1**: List of Sources for FAQs about Adolescent Idiopathic Scoliosis | |
| --- | --- |
| **1** | **American Family Phycisian** |
|  | <https://www.aafp.org/pubs/afp/issues/2020/0101/p19.html> |
| 1 | What Is the Clinical Significance of Adolescent Idiopathic Scoliosis? |
| 2 | Should Children Be Screened? |
| 3 | How Useful Is Physical Examination in Detecting Clinically Significant Scoliosis? |
| 4 | What Examination and/or Radiologic Findings Warrant Referral? |
| 5 | What Treatments Are Effective? |
| **2** | **Surgery for Adolescent Idiopathic Scoliosis Frequently Asked Questions** |
|  | [https://www.srs.org/Files/Patient-Brochures/Patient_AIS_ Surgery-handbook_FAQ.pdf](https://www.srs.org/Files/Patient-Brochures/Patient_AIS_Surgery-handbook_FAQ.pdf) |
| 1 | What should I bring with me to the hospital? |
| 2 | Do I need to do anything special before my surgery? |
| 3 | Should I take my regular medications before surgery? |
| 4 | What if I am having my period at the time of surgery? |
| 5 | What is done during the surgery? |
| 6 | Who will be in the operating room with me? |
| 7 | Can parents come into the operating room? |
| 8 | Will I wake up during the surgery? |
| 9 | Will I require a blood transfusion as a result of the surgery? |
| 10 | Is it possible to get my own blood back if I need a blood transfusion? |
| 11 | Do I need a plastic surgeon to close my incision? |
| 12 | Will I have pain after surgery? |
| 13 | How will my pain be controlled? |
| 14 | I’m concerned about the possibility of becoming addicted to narcotic pain medication. |
| 15 | Will I need a brace after surgery? |
| 16 | How long will I be in the hospital? |
| 17 | Will I be able to eat right after surgery? |
| 18 | Can I lie on my back after surgery? |
| 19 | How will I go to the bathroom after my surgery? |
| 20 | How will I get home? |
| 21 | What medicines will I take at home? |
| 22 | Will I be able to go up and down stairs at home? |
| 23 | Will I need a hospital bed or special mattress? |
| **3** | **Asian medical scientists** |
|  | [https://asiamedicalspecialists.hk/en/health-info/74/Juvenile-and-Adolescent- Idiopathic-Scoliosis-FAQs](https://asiamedicalspecialists.hk/en/health-info/74/Juvenile-and-Adolescent-Idiopathic-Scoliosis-FAQs) |
| 1 | What is scoliosis? |
| 2 | Are there different types of scoliosis? |
| 3 | How is scoliosis diagnosed? |
| 4 | Are there different types of Juvenile & Adolescent Idiopathic Scoliosis? |
| 5 | What causes Juvenile & Adolescent Idiopathic Scoliosis? |
| 6 | Tell me more about the genetics? |
| 7 | How likely is my child to be affected with Juvenile or Adolescent Idiopathic Scoliosis? |
| 8 | How do I know if my child has scoliosis? |
| 9 | How is scoliosis measured? |
| 10 | When does scoliosis become important? |
| 11 | How do we estimate remaining growth, and thus the likelihood of scoliosis progression? |
| 12 | Is scoliosis serious? |
| 13 | When does scoliosis require treatment? |
| 14 | What are the different kinds of operative treatment? |
| 15 | What are the complications of operative treatment? |
| 16 | What is the outcome of treatment of scoliosis? |
| **4** | **Boston Medical** |
|  | [https://www.bmc.org/orthopedic-surgery/pediatrics/scoliosis-children-and- adolescents/scoliosis-frequently-asked](https://www.bmc.org/orthopedic-surgery/pediatrics/scoliosis-children-and-adolescents/scoliosis-frequently-asked) |
| 1 | What is the difference between idiopathic scoliosis and other types of scoliosis? |
| 2 | How serious is adolescent scoliosis? |
| 3 | Does scoliosis cause back pain? |
| 4 | Can scoliosis curves get better on their own? |
| 5 | What can I do to prevent my scoliosis from getting worse? |
| 6 | Is it safe for my child to exercise and participate in sports? |
| 7 | Will my child be able to live a normal life? |
| 8 | Does scoliosis run in families? |
| 9 | If I have scoliosis, will my children have it? |
| 10 | Does my child's bad posture cause the scoliosis? |
| 11 | Does a leg length difference cause or worsen the curve? |
| 12 | Do sports activities or heavy backpacks cause scoliosis? |
| 13 | Is scoliosis related to an injury? |
| 14 | Could I have prevented scoliosis? |
| 15 | How early should children be screened for scoliosis? |
| 16 | Do siblings of children with scoliosis need to be checked? |
| 17 | When should the child of parents who have scoliosis be examined? |
| 18 | Why didn't we notice it sooner? |
| 19 | Why didn't our pediatrician see it sooner? |
| 20 | What health problems might I have later in life as a result of scoliosis? |
| 21 | Will I have a hump on my back when I get older? |
| 22 | One of my hips looks higher than the other. Can anything correct this? |
| 23 | Will having scoliosis affect my ability to have children? |
| 24 | Can I have an epidural in the future? |
| 25 | Does having scoliosis make me more prone to osteoporosis? |
| 26 | Will having scoliosis influence what I do later in life, such as what jobs I can do? |
| 27 | Will the metal detectors go off in airport security after I have rods placed in my spine? |
| 28 | If I have a spinal fusion, will I need antibiotics before dental work? |
| 29 | What scoliosis research is currently being conducted? |
| 30 | Are children born with idiopathic scoliosis? |
| 31 | Is there genetic testing for scoliosis? |
| **5** | **Adolescent idiopathic scoliosis for the primary care physician: frequently asked questions** |
|  | [https://www.researchgate.net/publication/329021648_Adolescent_idiopathic_ scoliosis_for_the_primary_care_physician_frequently_asked_questions](https://www.researchgate.net/publication/329021648_Adolescent_idiopathic_scoliosis_for_the_primary_care_physician_frequently_asked_questions) |
| 1 | What is the association between schoolbag usage and AIS? |
| 2 | What is the relationship between exercising and AIS? |
| 3 | How do women's health issues relate to AIS? |
| 4 | What is the adulthood prognosis for patients diagnosed with AIS during adolescence? |
| **6** | **Scoliosis care center** |
|  | <https://scoliosiscarecenters.com/faq/> |
| 1 | What is scoliosis? |
| 2 | What is Adolescent Idiopathic Scoliosis? |
| 3 | What Causes Adolescent Idiopathic Scoliosis? |
| 4 | Is Scoliosis Treatable? |
| 5 | Can Treating Scoliosis Make You Taller? |
| 6 | How Do You Treat Scoliosis? |
| 7 | What is Scoliosis Treatment? |
| 8 | When Does Scoliosis Need Treatment? |
| 9 | With Cases That are in Surgical Range, What are the Factors you Consider When Giving Advice? |
| 10 | How Safe is Scoliosis Surgery? |
| 11 | Does Scoliosis Surgery Hurt? |
| 12 | How Much Does Scoliosis Care Centers Treatment Cost? |
| 13 | Do We Treat Adults With Scoliosis? |
| 14 | How Soon Should We Start Noticing Permanent Changes? |
| 15 | How Many X-rays are Taken and Do you Use Lead Protection to Limit Exposure? |
| 16 | What are the Hotel/Accommodation Options if I am Attending Scoliosis Care  Centers? |
| 17 | Is Lunch Provided During Treatment? |
| 18 | Why Do You Use Hard Scoliosis Braces? Don’t They Make Scoliosis Worse? |
| 19 | What is Scoliosis Care Centers’ Success Rate? |
| 20 | What is the Largest Cobb Angle that Scoliosis Care Centers has Managed? |
| **7** | **British scoliosis society** |
|  | <https://britscoliosis.org.uk/Adolescent-Idiopathic-Scoliosis> |
| 1 | What is Adolescent Idiopathic Scoliosis? |
| 2 | How would I know I had scoliosis? |
| 3 | Why does it matter? |
| 4 | What should I do if I think I have scoliosis? |
| 5 | How will the doctors check if I have scoliosis? |
| 6 | How can you treat Adolescent Idiopathic Scoliosis? |
| **8** | **Scoliosis Reduction Center** |
|  | [https://www.scoliosisreductioncenter.com/blog/what-is-adolescent- idiopathic-scoliosis](https://www.scoliosisreductioncenter.com/blog/what-is-adolescent-idiopathic-scoliosis) |
| 1 | What is Adolescent Idiopathic Scoliosis? What Does It Mean? |
| 2 | What Does Idiopathic Scoliosis Mean? |
| 3 | Does Idiopathic Scoliosis Get Worse? |
| 4 | What is the Treatment for Adolescent Idiopathic Scoliosis? |
| 5 | Does Adolescent Idiopathic Scoliosis Go Away? |
| **9** | **HSS** |
|  | https://www.hss.edu/conditions_adolescent-idiopathic-scoliosis.asp |
| 1 | What is adolescent idiopathic scoliosis? |
| 2 | How is it diagnosed? |
| 3 | How is it treated? |
| 4 | How does scoliosis surgery work? |
| 5 | What are the results of spinal fusion surgery? |
| 6 | What is the expected recovery time? |
| 7 | Will the scoliosis curve of my nearly fully-grown 14-year-old child continue  to increase and become a problem later in life? |
| 8 | Why doesn't my doctor recommend electrical stimulation to correct scoliosis? |
| 9 | What are the risks of injury to my nervous system during scoliosis surgery? |
| 10 | What are the chances that a 15-year-old teenager who gets scoliosis surgery will need additional surgery later in life? |
| 11 | What are the advantages of an anterior versus a posterior scoliosis surgery? |
| **10** | **Scoliosis association UK** |
|  | <https://sauk.org.uk/types-of-scoliosis/adolescent-idiopathic-scoliosis/> |
| 1 | What is scoliosis? |
| 2 | Why has this happened? |
| 3 | I have just been diagnosed with scoliosis. What is going to happen now? |
| 4 | What’s the prognosis? |
| 5 | Could the scoliosis have been prevented? |
| 6 | Should I have noticed my child’s curve sooner? |
| 7 | Will scoliosis mean that my child can’t take part in sport? |
| 8 | I have seen a scoliosis specialist and I am still unclear as to what course of  treatment to have. |
| 9 | Will I have any problems with pregnancy? |
| 10 | My teenager is struggling emotionally with scoliosis what can I do? |
| 11 | How can I encourage my teenager to wear their brace? |
| 12 | How long does it take to recover from scoliosis (fusion) surgery? |
| 13 | I am not happy with how my child has been treated by the specialist or at the  hospital what should I do? |
| **11** | **WHAT INFORMATION DO TEENAGERS WITH IDIOPATHIC SCOLIOSIS AND THEIR  FAMILIES NEED WHEN FIRST DIAGNOSED?** |
|  | <https://cms.galenos.com.tr/Uploads/Article_48342/jtss-32-93-En.pdf> |
| 1 | What is scoliosis? |
| 2 | What causes scoliosis? |
| 3 | Is it hereditary? |
| 4 | Will it get better? |
| 5 | What happens now? |
| 6 | What treatment options are there for me? |
| 7 | Will I need an operation? |
| 8 | How will it affect me in later life? |
| **12** | **Healthy families** |
|  | [https://blogs.ohsu.edu/doernbecher/2017/06/28/your-scoliosis-questions -answered/](https://blogs.ohsu.edu/doernbecher/2017/06/28/your-scoliosis-questions-answered/) |
| 1 | What is scoliosis? |
| 2 | How is scoliosis diagnosed? |
| 3 | Why are girls more likely to get scoliosis? |
| 4 | What kind of treatments or procedures are available for patients with scoliosis? |
| 5 | What advice or information would you give someone who will have surgery  for scoliosis? |
| **13** | **FLEX BLOG** |
|  | <https://flexclinic.com.au/blog/adolescent-idiopathic-scoliosis-ais> |
| 1 | What is AIS? |
| 2 | How is AIS diagnosed? |
| 3 | What are the risks for progression of the curve? |
| 4 | How is scoliosis treated? |
| 5 | Which activities or movements should be avoided? |
| 6 | How does physiotherapy help with the scoliotic spine? |
| 7 | What is the effect of bracing on the scoliotic spine? |
| 8 | What are the major concerns of adolescents with scoliosis? |
| **14** | **ChatGPT-4 - most common questions about AIS** |
|  | <https://chat.openai.com/> |
| 1 | What is Adolescent Idiopathic Scoliosis (AIS)? |
| 2 | What causes AIS? |
| 3 | How is AIS diagnosed? |
| 4 | What are the signs and symptoms of AIS? |
| 5 | Is AIS hereditary? |
| 6 | What is the prevalence of AIS? |
| 7 | How does AIS progress? |
| 8 | What is the Cobb angle and why is it significant? |
| 9 | What are the treatment options for AIS? |
| 10 | What is the goal of treatment in AIS? |
| 11 | Are there non-surgical treatment options for AIS? |
| 12 | What types of braces are used for AIS and how effective are they? |
| 13 | What is the typical age for surgery and what does surgery entail? |
| 14 | What are the risks associated with surgery for AIS? |
| 15 | How will AIS affect daily life and physical activity? |
| 16 | Can physical therapy or exercises help in managing AIS? |
| 17 | How often should follow-up appointments be scheduled? |
| 18 | What is the long-term outlook for individuals with AIS? |
| 19 | Are there any complications associated with AIS in adulthood? |
| 20 | Are there support groups or resources for families dealing with AIS? |
