# Supplementary material for: Is the information provided by large language models valid in educating patients about adolescent idiopathic scoliosis? An evaluation of content, clarity, and empathy: The perspective of the European Spine Study Group
Source: Spine Deform. 2024 Nov 4;13(2):361–72. doi: 10.1007/s43390-024-00955-3 (PMC11893626; doi:10.1007/s43390-024-00955-3)
Supplement: Supplementary file 2 — Supplementary file2 (DOCX 38 KB) [file 43390_2024_955_MOESM2_ESM.docx]

**Supplementary Material 2:** Detailed answers of three LLMs to 10 common FAQs about Adolescent Idiopathic Scoliosis

**ChatGPT-3.5**

1. What distinguishes Adolescent Idiopathic Scoliosis from other scoliosis types, and are there different forms?

Answer:

"Adolescent Idiopathic Scoliosis (AIS) is a specific type of scoliosis that typically manifests during adolescence, primarily in girls. It's characterized by a sideways curvature of the spine, but what sets it apart from other scoliosis types are its key features:

Age of Onset: AIS typically develops during adolescence, between the ages of 10 and 18, whereas other types of scoliosis can occur at any age.

Idiopathic Nature: "Idiopathic" means the exact cause is unknown. AIS arises without a clear underlying reason, while other forms may be linked to conditions like congenital spinal abnormalities or neuromuscular disorders.

Gender Predilection: AIS is more common in females, with a female-to-male ratio of 10:1, whereas other forms may not show this gender bias.

Curve Patterns: AIS often presents as a single, "C"-shaped curve in the spine, typically in the thoracic (upper back) or lumbar (lower back) regions. Other forms may exhibit different curve patterns.

It's crucial to note that early detection and monitoring are vital for AIS. Treatment options, if necessary, can include bracing or surgery, depending on the severity and progression of the curvature. Regular check-ups and open communication with your healthcare team are key to addressing concerns and ensuring the best possible outcome."

2. How is AIS diagnosed, and what role do screening, imaging, and physical examination play in detecting it?

Answer:

"Adolescent Idiopathic Scoliosis (AIS) is typically diagnosed through a combination of screening, imaging, and physical examination:

Screening: AIS screening often begins in school settings, where nurses or trained healthcare professionals visually assess the child's back for any signs of curvature. However, it's essential to understand that this initial screening is not definitive and only serves as a preliminary step.

Physical Examination: A thorough physical examination by a healthcare provider, such as a pediatrician or orthopedic specialist, is crucial. They will assess the patient's posture, spinal alignment, and any signs of asymmetry. Specific tests, like the Adam's Forward Bend Test, can help identify potential scoliotic curvature.

Imaging: If there are concerns based on the physical examination or family history, X-rays are the gold standard for confirming and quantifying AIS. These images provide precise measurements of the spinal curvature, helping us determine the severity and treatment plan. MRI or CT scans may be necessary in specific cases to evaluate the spinal cord and nerves.

Early detection is vital, as AIS can progress rapidly during growth spurts. If diagnosed, we consider various factors like age, degree of curvature, and skeletal maturity to tailor a treatment plan. Treatment options range from observation for mild cases to bracing or surgery for more severe curvatures.

I recommend seeking a consultation with a spine specialist for a comprehensive evaluation if AIS is suspected. Your child's long-term spine health and well-being are our top priorities."

3. Can you summarize the treatment options for AIS and their indications and overall effectiveness?

Answer:

"Certainly, adolescent idiopathic scoliosis (AIS) is a condition where the spine curves sideways in a typically healthy adolescent without an underlying cause. Treatment options for AIS depend on the severity of the curvature and individual factors.

Observation: For mild curves, close monitoring with periodic X-rays is often recommended to ensure the curve doesn't progress significantly.

Bracing: Bracing is considered when the curve is moderate (25-40 degrees) and still growing. The type and duration of bracing depend on several factors, including the curve pattern and patient compliance.

Surgery: Surgery is considered when the curve reaches 45-50 degrees or more and continues to progress despite bracing. Spinal fusion surgery aims to straighten and stabilize the spine. The effectiveness is generally high in terms of curve correction but carries risks like infection and limited spinal flexibility.

The choice of treatment is individualized and takes into account factors like curve magnitude, skeletal maturity, and patient's lifestyle. The earlier the intervention, the better the chances of halting curve progression. Regular follow-ups are essential to monitor the condition. It's important for patients and parents to discuss treatment options thoroughly with their orthopedic spine specialist to make an informed decision based on their specific circumstances."

4. What are the primary causes of AIS, and could posture, sports, or carrying heavy items have contributed to it?

Answer:

"Adolescent Idiopathic Scoliosis (AIS) primarily has a genetic basis and tends to run in families, so it's not caused by poor posture, sports activities, or heavy lifting. The exact genetic factors responsible for AIS are still being researched, but it's crucial to understand that it's not a result of external factors or lifestyle choices.

AIS typically develops during adolescence when the spine is growing rapidly. While poor posture or carrying heavy items won't directly cause AIS, they can exacerbate existing curvature or discomfort. It's essential for adolescents to maintain good posture and avoid excessive strain on their backs, but these factors aren't the root causes of AIS.

As a spine surgeon, my goal is to provide comprehensive care for AIS patients. We focus on monitoring the curve's progression, considering non-surgical options such as bracing when necessary, and performing surgery only in severe cases. Early detection and regular follow-ups are crucial. Please remember that AIS isn't anyone's fault; it's a complex condition that requires medical attention and support. If you have concerns about your child's spine health, consult with a specialist for a personalized evaluation and guidance."

5. How is the progression of AIS estimated, especially in relation to my child's growth and how likely is it?

Answer:

"In adolescent idiopathic scoliosis (AIS), the progression can vary greatly among individuals. To estimate the likelihood of progression in relation to your child's growth, we consider several factors:

Age and Skeletal Maturity: AIS typically develops during adolescence when the spine is still growing. The risk of progression is higher during the growth spurt, which usually occurs just before puberty.

Cobb Angle: The degree of spinal curvature, measured by the Cobb angle on X-rays, is a key indicator. A Cobb angle of less than 25 degrees is often considered mild and has a lower risk of progression.

Gender: Girls have a higher likelihood of progression compared to boys, especially if they have a thoracic (upper back) curve.

Curve Type: The location and pattern of the curve can influence the risk. Thoracic curves tend to progress more than lumbar curves.

Family History: A family history of scoliosis may increase the risk of progression.

Bracing: If your child requires bracing, adherence to the treatment plan is crucial. Bracing can effectively halt progression in many cases.

Regular Monitoring: Periodic X-rays and clinical evaluations by a spine specialist are essential to track progression.

While AIS can progress, the majority of cases remain stable or have mild progression. Timely monitoring and early intervention, if necessary, can significantly reduce the risk of severe curvature. I recommend discussing your child's specific case with a spine specialist who can provide personalized guidance and address your concerns."

6. What restrictions on physical activity and future life events like pregnancy can we expect after scoliosis surgery?

Answer:

"After scoliosis surgery, it's crucial to understand that physical activity restrictions will vary depending on individual factors, such as the type of surgery, your overall health, and your surgeon's recommendations. In general, you can expect a gradual return to physical activities.

In the initial post-operative phase, there will be limitations. Activities like heavy lifting, strenuous sports, and twisting motions should be avoided for several months. Your surgeon will provide specific guidelines tailored to your case.

As for future life events like pregnancy, it's essential to discuss this with your surgical team. Scoliosis surgery typically does not directly impact pregnancy. However, the residual spinal hardware may cause discomfort during pregnancy, and you should inform your obstetrician about your surgical history. They can provide guidance on managing any potential discomfort.

Remember, the goal of scoliosis surgery is to improve your quality of life, and most patients can lead active, fulfilling lives after recovery. Open communication with your surgeon and following their guidance is key to a successful outcome."

7. What long-term outcomes should we anticipate for my child with AIS, including potential health issues and lifestyle impacts?

Answer:

"Adolescent idiopathic scoliosis (AIS) is a condition characterized by an abnormal curvature of the spine during adolescence. Long-term outcomes for your child with AIS can vary depending on the severity of the curvature and the success of treatment.

Spinal Health: Most individuals with AIS lead healthy lives with little to no impact on their overall health. Regular monitoring is essential to ensure the curve doesn't progress significantly.

Curve Progression: In some cases, the curvature may progress, potentially causing pain or discomfort. Monitoring during growth spurts is crucial as this is when progression is more likely.

Lifestyle: AIS typically doesn't limit lifestyle choices. Your child can participate in sports and activities as long as it's within their comfort and doesn't exacerbate symptoms.

Treatment: The approach may include observation, bracing, or surgery. Advances in surgical techniques have improved outcomes, and minimally invasive options reduce recovery time.

Emotional Well-being: It's important to support your child emotionally. AIS can affect self-esteem due to body image concerns.

Regular Follow-ups: Consistent follow-up with a spine specialist is vital to track progress and address any concerns promptly.

Future Pregnancy: If applicable, women with AIS can have successful pregnancies, but medical guidance is crucial to manage any potential discomfort.

Remember that each case is unique, and personalized care is essential. Early detection and a comprehensive treatment plan can significantly impact your child's long-term outcomes."

8. What does aftercare involve, and how often are follow-up visits needed post-surgery for AIS?

Answer:

"After surgery for Adolescent Idiopathic Scoliosis (AIS), the aftercare process is crucial for a successful outcome. Typically, you can expect a hospital stay of 3-5 days following surgery. During this time, our team will closely monitor your recovery, manage pain, and provide early mobility guidance. After discharge, a structured rehabilitation program is essential to regain strength and flexibility.

Follow-up visits are scheduled at regular intervals, with the first occurring around 2-4 weeks post-surgery. Subsequent visits are usually scheduled at 3, 6, and 12 months after surgery. These appointments serve to assess your progress, monitor spinal fusion healing, and ensure that any potential issues are promptly addressed.

It's important to note that the frequency and duration of follow-up visits may vary depending on individual factors and surgical techniques. Our aim is to ensure your spine stabilizes correctly, minimize complications, and address any concerns you or your parents may have. Communication with your healthcare team is key throughout this process to tailor your aftercare plan to your specific needs. We are here to support you on your journey to recovery, and our goal is your long-term spinal health and well-being."

9. Will surgery correct the cosmetic concerns of AIS, like uneven shoulders or back humps, and what can be done to minimize the visibility of their scar?

Answer:

"Certainly, adolescent idiopathic scoliosis (AIS) is a condition where the spine curves to the side, often causing uneven shoulders or a visible hump in the back. Surgery can be an effective option for correcting the cosmetic concerns associated with AIS. The main goal of surgery is to straighten the spine and restore a more natural alignment.

To minimize the visibility of the surgical scar, several techniques are employed. Surgeons typically use small incisions and minimally invasive procedures whenever possible. This helps reduce scarring and promotes faster recovery. Additionally, suturing techniques and adhesive dressings are used to optimize scar appearance.

It's essential to remember that while surgery can improve the cosmetic aspects of AIS, the decision to proceed should be based on a comprehensive evaluation of the curve's severity, the patient's age, and their overall health. Non-surgical options, such as bracing or physical therapy, may be considered first, depending on the individual case.

Ultimately, a thorough discussion with your surgeon, considering all aspects of AIS and the available treatment options, will help you make an informed decision that suits your specific needs and concerns."

10. What are the key symptoms and the clinical presentation of AIS, and is back pain a significant indicator?

Answer:

"Adolescent Idiopathic Scoliosis (AIS) primarily affects young individuals during their growth spurt years. Its key symptoms include:

Spinal Curvature: A noticeable sideways curvature of the spine is the hallmark of AIS. This may cause one shoulder or hip to appear higher than the other.

Asymmetry: Uneven shoulder blades or waistline can be observed.

Fatigue: Some individuals might experience fatigue or discomfort, especially during prolonged periods of sitting or standing.

Unequal Leg Length: In severe cases, leg length discrepancy can occur.

Notably, back pain is not typically a primary symptom of AIS. Most patients do not experience significant pain directly related to the curvature. However, occasional mild discomfort or muscle fatigue can be present, often due to postural changes. It's essential to distinguish between general back pain and AIS-related symptoms.

For patients and parents, early detection through routine screenings is crucial. Timely intervention can prevent progression and associated health issues. A thorough evaluation by a spine specialist, like myself, is essential to confirm the diagnosis and determine the most appropriate course of action, which may include observation, bracing, or, in severe cases, surgery. Always consult a healthcare professional for a precise assessment and personalized guidance."

**Chat GPT4.0**

1. What distinguishes Adolescent Idiopathic Scoliosis from other scoliosis types, and are there different forms?

Answer:

"Adolescent Idiopathic Scoliosis (AIS) is a condition characterized by a three-dimensional deformity of the spine that appears in late childhood or adolescence, typically between ages 10 to 18, without a clear underlying cause. It is distinguished from other types of scoliosis by its idiopathic nature, meaning it develops without a known cause, unlike congenital, neuromuscular, or syndromic scoliosis, which are associated with vertebral anomalies, neurological or muscular diseases, or syndromes, respectively.

AIS is categorized based on the curvature of the spine measured by the Cobb angle: mild (less than 20 degrees), moderate (20 to 40 degrees), and severe (greater than 40 degrees). Treatment varies depending on the severity and progression risk, ranging from observation for mild cases to bracing for moderate cases, and potentially surgery for severe or progressive curvatures.

Recent advancements in the understanding of AIS suggest a multifactorial etiology, including genetic and environmental factors. Innovations in treatment include growth modulation techniques and advanced surgical methods that aim to preserve spinal mobility. Parents and patients often have concerns about the impact on daily life, long-term health, and treatment options. Timely diagnosis and individualized treatment planning are crucial to optimize outcomes and address these concerns."

2. How is AIS diagnosed, and what role do screening, imaging and physical examination play in detecting it?

Answer:

"Adolescent Idiopathic Scoliosis (AIS) is diagnosed through a multifaceted approach that typically begins with a screening process. During screening, health professionals look for visual signs of scoliosis such as asymmetry in shoulder height, scapular prominence, or waistline. This is often conducted in school settings or during routine health check-ups.

If scoliosis is suspected, a physical examination is performed. The Adam’s forward bend test is a key component, where the patient bends forward at the waist, and the examiner looks for any unevenness in the rib cage or lower back, which can indicate spinal curvature.

Imaging plays a critical role; the standard is an X-ray of the full spine, taken from the back and the side. The Cobb angle, measured on the X-ray, quantifies the curvature and aids in classifying the severity of scoliosis. Advanced imaging techniques, like MRI, may be used if neurological symptoms are present or to evaluate the spinal cord.

Early detection through screening and timely evaluation with physical examination and imaging are pivotal in the management of AIS, as they allow for early intervention and monitoring of the condition's progression. This multi-tiered diagnostic approach is essential to devise an appropriate treatment strategy and inform patients and parents effectively."

3. Can you summarize the treatment options for AIS and their indications and overall effectiveness?

Answer:

"Adolescent Idiopathic Scoliosis (AIS) is a curvature of the spine with no clear underlying cause, prevalent in patients aged 10-18. Treatment modalities are tailored based on the severity of the curve, the risk of progression, and the patient's stage of skeletal maturity.

Non-surgical options include observation for curves less than 20 degrees in skeletally immature individuals. Back braces are indicated for curves between 20-40 degrees to prevent progression during growth spurts. The effectiveness of bracing correlates with adherence to prescribed wear times.

Physical therapy can be adjunctive to improve posture and musculoskeletal conditioning, but it does not alter curve progression. Schroth method exercises, designed for scoliosis, aim to realign and mobilize the spine, although evidence for their efficacy is mixed.

Surgical intervention is generally reserved for curves exceeding 40-50 degrees or for curves that progress despite bracing, due to the risk of future health problems. The main surgical approach is spinal fusion, which has high success rates in halting curve progression and maintaining correction, albeit with risks inherent to major surgery.

Recently, vertebral body tethering (VBT), a less invasive surgical option allowing for spinal growth and mobility preservation, has shown promise in select patients, but long-term data on its effectiveness is still being gathered.

It's crucial for patients and parents to understand that while treatment can effectively manage AIS, monitoring by a spine specialist is essential throughout the patient's growth period to ensure the best outcome."

4. What are the primary causes of AIS, and could posture, sports, or carrying heavy items have contributed to it?

Answer:

"Adolescent idiopathic scoliosis (AIS) is a condition characterized by a curvature of the spine with unknown etiology, predominantly affecting those between 10-18 years of age. The term 'idiopathic' indicates that the precise causes are not fully understood. Research suggests a multifactorial causation with genetic, hormonal, and environmental factors potentially playing roles. A significant body of evidence points to hereditary patterns, indicating a genetic predisposition to the condition.

Contrary to common concerns, everyday activities such as sports, carrying heavy items, or postural habits have not been substantiated as causes of AIS. While these factors may influence back pain or other orthopedic conditions, they do not cause the spinal curvature associated with AIS. The condition is not preventable, and its onset is not due to actions or habits of adolescents.

It is important for patients and parents to understand that AIS is not caused by anything the adolescent did or did not do. Treatment focuses on the severity of the curve, skeletal maturity, and potential for progression, with options ranging from observation, bracing, to surgical intervention in more severe cases. Regular follow-up with a specialist is crucial for monitoring and managing the condition effectively."

5. How is the progression of AIS estimated, especially in relation to my child's growth and how likely is it?

Answer:

"Adolescent idiopathic scoliosis (AIS) progression is closely linked to growth spurts, with the most significant risk occurring during the peak velocity of growth typically observed in adolescence. The Risser sign, which assesses the ossification of the iliac apophysis, is a traditional radiographic marker used to estimate the stage of skeletal maturity and thus the potential for progression of the curvature. A lower Risser grade (0-2) usually indicates a higher risk of curve progression due to more remaining growth.

In contemporary practice, the maturity of the patient is also gauged through less invasive methods such as the Tanner stage, which examines pubertal development, and hand and wrist radiographs to evaluate bone age. More recent advancements include the use of genetic testing, which can predict the likelihood of progression to a severe curve.

The probability of progression also depends on the magnitude of the curve at the time of diagnosis; curves less than 20 degrees are less likely to progress, whereas those greater than 30 degrees have a higher risk, especially if substantial growth remains. Treatment decisions are based on these assessments and may range from observation to bracing or surgery, with the goal of minimizing progression while maximizing quality of life.

Parents should engage with a spine specialist to monitor their child's growth and scoliosis regularly to adjust treatment plans as needed and to provide support throughout the management of AIS."

6. What restrictions on physical activity and future life events like pregnancy can we expect after scoliosis surgery?

Answer:

"Adolescent idiopathic scoliosis (AIS) surgery primarily aims to correct spinal deformity and prevent its progression. Postoperatively, physical activity restrictions are usually recommended during the initial healing phase, which can last from 3 to 6 months. During this period, activities that could risk a fall or impact the spine are typically avoided to ensure proper fusion and healing of the spine.

After the initial healing phase, most patients are encouraged to gradually return to normal activities, including sports, with some precautions tailored to the individual's surgery and recovery status. High-impact activities or contact sports may require a longer waiting period or be discouraged depending on the spinal fusion's extent and the patient’s overall condition.

Regarding future life events like pregnancy, most women who have undergone scoliosis surgery can expect to have normal pregnancies and deliveries. However, it's essential to have an obstetrician and spine specialist collaborate on care during pregnancy. A cesarean section is not necessarily required, and many can have vaginal deliveries unless other obstetric factors necessitate a C-section.

It's crucial for patients and parents to discuss specific concerns and expectations with their surgeon, as recommendations can vary based on individual surgical techniques, the extent of fusion, and the patient's overall health and lifestyle. Regular follow-up is important to monitor the spine's stability and adapt recommendations as needed."

7. What long-term outcomes should we anticipate for my child with AIS, including potential health issues and lifestyle impacts?

Answer:

"Adolescent Idiopathic Scoliosis (AIS) is typically managed with monitoring, bracing, or surgical interventions depending on the severity and progression of the curve. Long-term outcomes vary, but with early and appropriate treatment, most individuals live healthy, active lives.

Monitoring is crucial. Mild curves often require no treatment other than regular follow-ups to ensure the curve does not worsen. Bracing is used to prevent curve progression in growing adolescents and is effective in many cases, especially when worn as prescribed.

For moderate to severe curves or those that progress rapidly, surgery may be considered. Modern surgical techniques like spinal fusion can significantly correct the curve and prevent further progression. Minimally invasive methods and advances in instrumentation have improved recovery times and outcomes.

After treatment, regular physical activity is encouraged. Most individuals can return to their pre-diagnosis activity level. Long-term, AIS patients generally do not have increased health issues, although some may experience mild back pain or cosmetic concerns related to their spine's appearance.

Psychosocial aspects are important. Adolescents may experience body image issues or anxiety related to their condition or treatment. Support from family, peers, and counseling can be beneficial.

It's essential to discuss your child’s specific case with a spine specialist to get personalized advice, as individual outcomes can vary based on the curve severity, treatment type, and adherence to medical recommendations."

8. What does aftercare involve, and how often are follow-up visits needed post-surgery for AIS?

Answer:

"Aftercare for adolescent idiopathic scoliosis (AIS) post-surgery is crucial for optimal recovery. It usually involves a combination of rest, physical therapy, and follow-up appointments. The initial weeks after surgery require limited activity, with a gradual increase in mobility as tolerated. Pain management is a key component, typically involving medications and techniques to reduce discomfort.

Physical therapy plays a vital role, starting with light activities and progressing to more strenuous exercises over weeks to months. The goal is to strengthen the back and abdominal muscles to support the spine, improve flexibility, and enhance overall function. Patients are generally advised to avoid sports and activities that jar or twist the spine for several months post-surgery.

Follow-up visits are scheduled to monitor the healing process and the integrity of the spinal fusion. These visits are typically frequent in the first year—often at 2 weeks, 3 months, 6 months, and 12 months post-operation. After the first year, annual visits may be sufficient unless complications arise. Imaging tests, like X-rays or MRI scans, are performed during these visits to ensure the spine is healing correctly and the hardware remains properly positioned.

It's essential for patients and parents to maintain open communication with their healthcare team, promptly addressing any concerns such as pain, changes in neurological function, or signs of infection. Each patient's recovery timeline may vary, so aftercare is personalized to the individual's needs."

9. Will surgery correct the cosmetic concerns of AIS, like uneven shoulders or back humps, and what can be done to minimize the visibility of their scar?

Answer:

"Adolescent idiopathic scoliosis (AIS) is the most common form of scoliosis occurring during the growth spurt around puberty. Surgical intervention, typically recommended when the curvature exceeds 45-50 degrees, aims to correct the deformity, prevent progression, and address cosmetic concerns.

The primary surgical treatment, posterior spinal fusion, can significantly improve cosmetic issues like uneven shoulders and back humps. By realigning and stabilizing the spine, it restores balance and symmetry to the back's appearance. However, individual outcomes can vary based on the severity and flexibility of the curve, age, and surgical technique.

Minimizing scar visibility is also a consideration. Advanced minimally invasive techniques can reduce the size and appearance of scars. Post-operative care is crucial; silicone gel or sheeting, along with sun protection, can promote healing and lessen scar tissue's prominence. Dermatological consultation for scar management may be beneficial, as treatments like laser therapy and microneedling have shown promising results in scar appearance improvement.

It's essential for patients and parents to discuss their expectations and concerns with their surgeon, as they can provide tailored advice and set realistic outcomes for the postoperative appearance."

10. What are the key symptoms and the clinical presentation of AIS, and is back pain a significant indicator?

Answer:

"Adolescent idiopathic scoliosis (AIS) is the most common form of scoliosis, usually presenting during the growth spurt just before puberty. Key symptoms include a visible curvature of the spine when viewed from behind, shoulders or hips that appear uneven, and in some cases, the presence of a rib hump when the child bends forward. Unlike in adults, back pain is not typically a prominent symptom in AIS; however, it can be present in some adolescents.

Clinically, AIS is diagnosed with a physical examination and confirmed with imaging, such as an X-ray to assess the degree of spinal curvature, known as the Cobb angle. The Scoliosis Research Society endorses the Scoliosis Screening Guidelines to assist in early identification. Regular monitoring is vital, especially during peak growth periods, as progression can be rapid.

For most adolescents with this condition, the physical appearance of the curvature is the primary concern. Back pain, when it occurs, is usually mild but should be evaluated to rule out other causes. Treatment options range from observation for mild cases, to bracing for moderate curves, to surgical intervention for severe curvatures or rapidly progressing cases. Early detection and treatment are crucial for managing AIS effectively and minimizing its impact on quality of life."

**Google Bard**

1. What distinguishes Adolescent Idiopathic Scoliosis from other scoliosis types, and are there different forms?

Answer:

"Adolescent Idiopathic Scoliosis (AIS): Understanding the Distinctions

Adolescent idiopathic scoliosis (AIS) is the most common type of scoliosis, accounting for about 80% of all scoliosis cases. It is a sideways curvature of the spine that typically develops during the growth spurt just before puberty. AIS is considered idiopathic because the exact cause is unknown.

Distinguishing Features of AIS:

Age of onset: AIS typically develops between the ages of 10 and 18.

Gender predilection: AIS is more common in girls than boys, with a ratio of about 8:1.

Curve pattern: AIS curves are usually in an "S" or "C" shape and occur most often in the middle or lower part of the spine.

Types of AIS:

Lenke: This classification system is based on the location and severity of the curve. There are six Lenke types, ranging from mild to severe.

King: This classification system is based on the age of onset and the severity of the curve. There are five King types, ranging from mild to severe.

Addressing Patient and Parent Concerns:

Progression: The main concern for patients and parents is whether the curve will progress. While some curves stabilize over time, others may continue to worsen. Early detection and regular monitoring are crucial for managing AIS.

Treatment: Treatment options for AIS vary depending on the severity of the curve and the patient's age. Observation, bracing, and surgery are the main treatment options.

Psychological impact: AIS can have a significant psychological impact on adolescents, particularly girls. It is important to provide emotional support and help patients develop coping mechanisms.

Seeking Expert Guidance:

If you or your child has been diagnosed with AIS, it is important to consult with an experienced spine surgeon to discuss the best treatment options. Early intervention can help prevent curve progression and minimize the long-term impact of AIS."

2. How is AIS diagnosed, and what role do screening, imaging and physical examination play in detecting it?

Answer:

"Diagnosing Adolescent Idiopathic Scoliosis (AIS): A Comprehensive Approach

Early detection of adolescent idiopathic scoliosis (AIS) is crucial for managing the condition and preventing curve progression. A comprehensive diagnostic approach involves screening, imaging, and physical examination.

Screening:

School screenings: Scoliosis screening programs are often implemented in schools to identify children with potential spinal curvatures. These screenings typically involve forward bending tests and visual observations.

Family history: If you have a family history of AIS, it is important to inform your child's pediatrician so they can monitor your child's development closely.

Imaging:

X-rays: X-rays provide a detailed picture of the spine, allowing doctors to measure the severity of the curve and determine its location.

EOS imaging: EOS imaging is a specialized type of X-ray that provides full-length, low-dose images of the spine. It is particularly useful for assessing scoliosis curves in full detail.

Physical examination:

Visual observation: Doctors will observe the patient's posture and back shape to look for signs of scoliosis, such as uneven shoulders, prominent ribs, or a tilted pelvis.

Adams forward bend test: In this test, the patient bends forward at the waist while keeping their arms straight down. Doctors will observe the symmetry of the back and the presence of any bumps or curves.

Full-body examination: Doctors will also assess the patient's overall health and development, including checking for any neurological or musculoskeletal issues.

Addressing Patient and Parent Concerns:

Early detection: Early detection of AIS is essential for effective treatment and management. If you have concerns about your child's posture or back shape, consult with your pediatrician or an orthopedic specialist.

Regular monitoring: Regular monitoring is crucial for tracking curve progression and determining the need for treatment. Your doctor will recommend an appropriate monitoring schedule based on your child's individual case.

Open communication: Open communication between patients, parents, and healthcare providers is essential for managing AIS effectively. Ask questions, express concerns, and work together to develop a personalized treatment plan."

3. Can you summarize the treatment options for AIS and their indications and overall effectiveness?

Answer:

"Treatment Options for Adolescent Idiopathic Scoliosis (AIS): Tailored Approaches for Optimal Outcomes

Adolescent idiopathic scoliosis (AIS) is a prevalent spinal condition characterized by a sideways curvature of the spine. Treatment options for AIS vary depending on the severity of the curve, the patient's age, and individual preferences. Here's a summary of the main treatment options and their indications:

Observation:

For curves that are mild (less than 25 degrees) and not expected to progress, observation may be sufficient.

Regular monitoring is crucial to track curve progression and determine the need for intervention.

Bracing:

Bracing is a non-invasive treatment option for curves between 25 and 45 degrees.

Braces are typically worn for 20-23 hours per day to help prevent curve progression.

Bracing is most effective when started early, during the growth spurt just before puberty.

Surgery:

Surgery is recommended for curves that are severe (45 degrees or more) or that are rapidly progressing.

There are various surgical techniques available, including spinal fusion and instrumentation.

Spinal fusion involves permanently joining two or more vertebrae together to halt curve progression.

Instrumentation involves using metal rods, screws, and hooks to straighten the spine and maintain its alignment.

Overall Effectiveness:

With appropriate treatment, most patients with AIS can manage their condition and live healthy, active lives.

Observation is effective in preventing curve progression in mild cases.

Bracing is highly effective in preventing curve progression in moderate cases.

Surgery is highly effective in correcting severe curves and preventing further progression.

Addressing Patient and Parent Concerns:

Individualized treatment plans: Treatment decisions are made on a case-by-case basis, considering the patient's individual curve characteristics, age, and lifestyle.

Open communication: Patients and parents are encouraged to openly communicate their concerns and preferences with their healthcare providers.

Psychosocial support: Managing AIS can be emotionally challenging, and psychosocial support services can be beneficial for patients and their families.

Conclusion:

With a range of effective treatment options available, patients with AIS can achieve positive outcomes and maintain a high quality of life. Early detection, regular monitoring, and collaboration with healthcare providers are key to successful management of this condition."

4. What are the primary causes of AIS, and could posture, sports, or carrying heavy items have contributed to it?

Answer:

"Understanding the Causes of AIS: Dispelling Common Myths

Adolescent idiopathic scoliosis (AIS) is the most common type of scoliosis, affecting approximately 3% of adolescents. While the exact cause of AIS remains unknown, research suggests it is a complex interplay of genetic, hormonal, and environmental factors.

Primary Causes of AIS:

Genetics: Studies indicate that AIS has a strong genetic component. About 30% of AIS cases have a family history of the condition.

Hormonal changes: Hormonal changes during puberty are thought to play a role in AIS development. Estrogen, a female sex hormone, has been linked to increased curve progression.

Other factors: Other factors that may contribute to AIS include connective tissue abnormalities, neurologic imbalances, and biomechanical factors.

Addressing Posture, Sports, and Heavy Lifting Concerns:

Posture: While poor posture may not directly cause AIS, it can make the curvature more noticeable. Maintaining good posture is important for overall health and can help prevent other musculoskeletal problems.

Sports: Participation in sports does not increase the risk of developing AIS. In fact, regular exercise can be beneficial for overall health and muscle development.

Carrying heavy items: Carrying heavy items may cause temporary discomfort but does not contribute to AIS development. Proper lifting techniques are important to prevent back strain and injuries.

Conclusion:

AIS is a complex condition with multiple contributing factors. While there is no definitive cause-and-effect relationship between posture, sports, or heavy lifting and AIS development, maintaining good posture, engaging in regular exercise, and practicing proper lifting techniques are beneficial for overall health and well-being."

5. How is the progression of AIS estimated, especially in relation to my child's growth and how likely is it?

Answer:

"Estimating the progression of adolescent idiopathic scoliosis (AIS) is crucial for determining the appropriate treatment plan and monitoring the condition over time. Several factors are considered when estimating AIS progression, including the severity of the curve at diagnosis, the patient's age, and their growth spurt status.

Factors Affecting AIS Progression:

Curve Severity at Diagnosis: The severity of the curve at diagnosis is a significant predictor of progression. Curves that are more severe at diagnosis are more likely to progress than mild curves.

Patient's Age: Age plays a crucial role in AIS progression. Curves are more likely to progress during the growth spurt just before puberty. Once skeletal maturity is reached, curve progression is less common.

Growth Spurt Status: Monitoring the patient's growth spurt is essential for predicting AIS progression. Curves are more likely to progress during the peak growth spurt velocity, which typically occurs between 11 and 13 years of age for girls and 13 to 15 years of age for boys.

Methods for Estimating AIS Progression:

Observation and Monitoring: Regular observation and monitoring of the curve through physical examinations and X-rays are essential for tracking progression.

Risser Signs: Risser signs are radiographic indicators of skeletal maturity. As skeletal maturity progresses, the risk of curve progression decreases.

Prediction Models: Various prediction models have been developed to estimate the likelihood of curve progression based on factors such as curve severity, age, and Risser signs.

Addressing Patient and Parent Concerns:

Individualized Assessments: AIS progression is unique to each patient, and individualized assessments are crucial for accurate predictions.

Regular Monitoring: Regular monitoring allows for early detection of curve progression and timely intervention if necessary.

Open Communication: Open communication between patients, parents, and healthcare providers is essential for managing AIS effectively.

Conclusion:

Estimating AIS progression is a complex process that involves considering various factors and using multiple methods. Early detection and regular monitoring are crucial for managing AIS effectively and preventing potential complications."

6. What restrictions on physical activity and future life events like pregnancy can we expect after scoliosis surgery?

Answer:

"Physical Activity and Future Life Events After Scoliosis Surgery: Understanding the Impact and Embracing a Healthy Lifestyle

Adolescent idiopathic scoliosis (AIS) surgery can significantly improve spinal alignment and reduce curve progression, allowing individuals to lead active and fulfilling lives. However, it is important to understand the potential impact on physical activity and future life events like pregnancy.

Physical Activity Restrictions:

Early post-surgery period: Initially, patients are advised to avoid strenuous activities and heavy lifting to allow the surgical incisions to heal and the spine to stabilize.

Gradual return to activities: After a few weeks of recovery, patients can gradually resume physical activities, starting with low-impact exercises like walking, swimming, and cycling.

Individualized recommendations: Specific activity restrictions and timelines for returning to sports or high-impact activities are based on the individual's surgical procedure, curve severity, and recovery progress.

Consult with healthcare provider: Regular communication with your surgeon or physical therapist is essential to ensure a safe and appropriate return to physical activities.

Pregnancy and Scoliosis Surgery:

No increased risk of complications: Scoliosis surgery does not increase the risk of complications during pregnancy or childbirth.

Regular prenatal care: Regular prenatal care is crucial for monitoring both maternal and fetal health throughout pregnancy.

Delivery options: Delivery options for women with scoliosis surgery depend on various factors, including the severity of the curve and the individual's preference.

Consult with obstetric care provider: Consultation with your obstetric care provider is essential to discuss the best delivery options and ensure a safe and healthy pregnancy.

Conclusion:

Scoliosis surgery can significantly improve spinal alignment and enhance quality of life. With proper guidance and individualized recommendations, patients can lead active lifestyles and experience fulfilling pregnancies. Open communication with healthcare providers is key to navigating physical activity restrictions and pregnancy considerations."

7. What long-term outcomes should we anticipate for my child with AIS, including potential health issues and lifestyle impacts?

Answer:

"Long-Term Outcomes for Adolescents with AIS: Embracing a Healthy and Active Future

Adolescent idiopathic scoliosis (AIS) is a common condition affecting the spine, but with proper management, most individuals can lead healthy and fulfilling lives. Long-term outcomes for individuals with AIS vary depending on the severity of the curve, treatment interventions, and individual lifestyle choices.

Physical Health Outcomes:

Pain: Some individuals with AIS may experience mild to moderate pain, particularly in the back or neck. Maintaining good posture, engaging in regular exercise, and using pain management techniques can help alleviate discomfort.

Cardiopulmonary function: Severe AIS curves can affect lung and heart function. However, with early detection and appropriate treatment, most individuals do not experience significant cardiopulmonary complications.

Neurological function: Neurological complications are rare in AIS patients. However, regular monitoring for any signs of neurological involvement, such as numbness or tingling in the extremities, is important.

Lifestyle Impacts:

Physical activity: Most individuals with AIS can participate in a wide range of physical activities, including sports and recreational pursuits. However, it is crucial to listen to one's body and choose activities that are appropriate for their individual curve severity and treatment plan.

Self-esteem and body image: AIS can affect self-esteem and body image, especially during adolescence. Open communication with family, friends, and healthcare providers, as well as participation in support groups, can help individuals develop a positive body image and self-esteem.

Social interactions and relationships: AIS typically does not hinder social interactions or relationships. Encouraging participation in social activities and extracurricular pursuits can help individuals maintain a healthy social life.

Conclusion:

With proper management and a healthy lifestyle, individuals with AIS can enjoy a fulfilling life with minimal long-term health issues and lifestyle impacts. Regular monitoring, open communication with healthcare providers, and a positive attitude are key to navigating the challenges and achieving optimal outcomes."

8. What does aftercare involve, and how often are follow-up visits needed post-surgery for AIS?

Answer:

"Post-Surgical Care for AIS: Ensuring a Smooth Recovery and Optimal Outcomes

Following scoliosis surgery, comprehensive post-surgical care is crucial for a smooth recovery, optimal healing, and long-term management of the condition. This care plan includes regular follow-up visits, physical therapy, and lifestyle modifications.

Follow-up Visits:

Initial post-surgery visits: Frequent follow-up visits are scheduled in the initial weeks following surgery to monitor incision healing, assess neurological function, and check for any complications.

Regular monitoring: Regular follow-up visits are typically scheduled every few months to monitor curve progression, assess overall health, and address any concerns or questions.

X-rays: X-rays are typically taken at follow-up visits to evaluate the alignment of the spine and assess the fusion process.

Physical Therapy:

Post-operative rehabilitation: Physical therapy is an integral part of post-surgical care. It helps regain strength, flexibility, and range of motion in the back and surrounding muscles.

Individualized exercises: Physical therapists design personalized exercise programs tailored to the patient's individual needs and recovery progress.

Home exercise routine: Patients are instructed on home exercises to continue strengthening and maintaining their progress.

Lifestyle Modifications:

Pain management: Pain management techniques, such as over-the-counter pain relievers, ice packs, and heat therapy, can help alleviate discomfort during the recovery period.

Rest and activity modification: Patients are advised to rest adequately and avoid strenuous activities or heavy lifting for several weeks following surgery.

Nutrition and hydration: Maintaining a healthy diet and adequate hydration supports healing and overall well-being.

Conclusion:

Post-surgical care plays a critical role in ensuring a successful recovery from AIS surgery. Regular follow-up visits, personalized physical therapy, and appropriate lifestyle modifications contribute to optimal healing, curve management, and long-term spinal health."

9. Will surgery correct the cosmetic concerns of AIS, like uneven shoulders or back humps, and what can be done to minimize the visibility of their scar?

Answer:

"Addressing Cosmetic Concerns in AIS Surgery: Achieving a Balance Between Function and Appearance

Adolescent idiopathic scoliosis (AIS) surgery primarily aims to correct the curvature of the spine and prevent further progression. While the primary focus is on functional improvement, the surgery can also address cosmetic concerns, such as uneven shoulders or back humps.

Cosmetic Impact of AIS Surgery:

Spine alignment: Successful scoliosis surgery can significantly improve the alignment of the spine, reducing the appearance of uneven shoulders, back humps, or a tilted pelvis.

Scar management: Incisions from scoliosis surgery are typically located along the back, but they can be minimized using minimally invasive techniques and careful wound closure.

Scar camouflage: Various scar management techniques, such as silicone tapes, creams, and laser treatments, can help minimize the visibility of scars over time.

Individualized Approach:

Preoperative discussion: Discussing cosmetic concerns with your surgeon during the preoperative consultation is crucial. They can assess your individual curve and explain the expected cosmetic improvements.

Surgical techniques: The surgeon will select the surgical technique that best addresses both the functional and cosmetic goals of the procedure.

Postoperative care: Proper postoperative care, including scar management techniques, can help minimize the visibility of scars and optimize cosmetic outcomes.

Conclusion:

Scoliosis surgery can significantly improve the appearance of the spine and address cosmetic concerns related to AIS. Open communication with your surgeon, individualized treatment planning, and meticulous postoperative care are key to achieving a balance between function and aesthetics."

10. What are the key symptoms and the clinical presentation of AIS, and is back pain a significant indicator?

Answer:

"Key Symptoms and Clinical Presentation of Adolescent Idiopathic Scoliosis (AIS): Unveiling the Signs

Adolescent idiopathic scoliosis (AIS) is the most common type of scoliosis, affecting approximately 3% of adolescents. It is a sideways curvature of the spine that typically develops during the growth spurt just before puberty. AIS can present with various symptoms and clinical signs, but back pain is not always a significant indicator.

Common Symptoms:

Visible curvature of the spine: A noticeable curvature of the spine, often observed when bending forward, may indicate AIS.

Uneven shoulders or hips: Uneven shoulders or hips, where one side appears higher than the other, can be a sign of scoliosis.

Prominent ribs: One side of the rib cage may appear more prominent or jutting out than the other.

Tilted pelvis: The pelvis may appear tilted to one side, causing one leg to appear shorter than the other.

Clinical Presentation:

Adams forward bend test: In this test, the patient bends forward at the waist while keeping their arms straight down. The doctor observes the symmetry of the back and the presence of any bumps or curves.

Full-body examination: The doctor will assess the patient's overall health and development, including checking for any neurological or musculoskeletal issues.

Role of Back Pain:

Not a consistent symptom: Back pain is not a consistent symptom of AIS. Many individuals with AIS do not experience any back pain, while others may have mild to moderate discomfort.

Varying severity: The severity of back pain can vary among individuals with AIS and may not correlate with the severity of the curve.

Other causes: Back pain can also result from other factors, such as poor posture, muscle strain, or underlying medical conditions.

Conclusion:

AIS can manifest with various symptoms and clinical signs, but back pain is not always a reliable indicator. Early detection and regular monitoring are crucial for managing AIS effectively. Consulting with an orthopedic specialist is essential to determine the appropriate treatment plan based on the individual's curve severity, symptoms, and overall health."
